# Sustainable Recycling Techniques of Pavement Materials

**DOI:** 10.3390/ma15248710

**Published:** 2022-12-07

**Authors:** Jiaqing Wang, Qiang Li, Kaijian Huang, Dongdong Ge, Fangyuan Gong

**Affiliations:** 1 College of Civil Engineering, Nanjing Forestry University, Nanjing 210037, China; 2 National Engineering Laboratory of Highway Maintenance Technology, Changsha University of Science & Technology, Changsha 410114, China; 3 School of Civil and Transportation Engineering, Hebei University of Technology, Tianjin 300401, China

Innovative sustainable techniques for transportation infrastructure enhancement have been proposed in recent decades. The recycling of solid wastes or used pavement materials is facing some challenges that are mainly related to reduced performance since the aging effect or other damages accumulated during the service period. Compared with normal pavement materials prepared with natural resources, the mechanical strength, chemical composition, and microstructure are obviously affected. Not only the raw materials but also the energies consumed during the life-cycle could be saved with recycled materials and improved durability by utilizing these innovative sustainable techniques.

This Special Issue attracted innovative and efficient techniques and materials for pavement recycling and reconstruction. The collected studies include pavement recycling techniques, the effective utilization of industrial and construction waste in infrastructure engineering, improvements in sustainable techniques for pavement materials, green low-carbon and durable pavement structures and materials, the smart construction (3D printing) of sustainable infrastructures, the investigation of durability performance enhancement by recycled pavement materials, and the application of life-cycle assessment (LCA) in evaluating infrastructure sustainability. 

The innovative green materials, new techniques, and main contributions of these studies are summarized below:

Xue et al. investigated the performance of an asphalt binder and mixture modified by lignin and waste engine oil [1]. Lignin, as a by-product or waste material of biomass materials, is used as a modifier for asphalt materials; similarly, waste motor oil is the non-distillable part collected from the solid–liquid copolymer formed by the consumption of motor oil after oxidation, etc. Waste motor oil and asphalt have relatively similar compositions and good recyclable value and are also considered to be good asphalt modifiers, both of which can be utilized and have great potential for utilization. In this study, two additives, lignin and waste engine oil, were used for matrix asphalt modification (previously, only a single additive was used to improve one aspect of the asphalt properties) and based on the Brookfield viscometer, dynamic shear rheometer, bending beam rheometer, rutting test, and low-temperature bending beam test, to explore the high- and low-temperature lignin-waste-engine-oil-modified asphalt binder and its mixes performance. The lignin increases the rotational viscosity, while waste oil decreases it. The lignin increases the elastic component, while waste motor oil decreases the elastic component. Waste motor oil can improve low-temperature performance. The dynamic stability obtained for asphalt mixtures has a good linear relationship with viscosity. 

Plastics are synthetic materials mainly derived from refined crude oil petroleum products; the main sources of waste plastics in the territory are plastic containers, plastic packaging, and other common plastic industrial products. Due to the huge amount of plastic production and the need to reduce environmental concerns, waste plastics are attractive for asphalt modification, and there are great prospects for development [2]. Using waste plastics as an asphalt modifier expands the application field of waste plastics, which solves the problem of waste plastic disposal and reduces environmental pollution. According to the source of waste plastics, choose a suitable processing process. When used for asphalt modification, scrap plastics can be made in various forms and sizes depending on the specific application. Asphalt modified with waste plastics alone is a physical modification. LCA methods can effectively quantify the environmental impact of waste plastics on asphalt and asphalt mix modification. The high-temperature performance of waste plastic-modified asphalt is quite effective after the introduction of such plastics. However, the low-temperature performance still needs to be improved in future studies. 

Municipal solid waste (MSW) is largely treated using incineration, and the resulting product is divided into fly ash and bottom ash, depending on where it is collected. Fly ash has long been used as a road construction material. Using fly ash in asphalt mixes and concrete structures reduces the requirement to use natural aggregates and mineral fillers [3]. Zhao et al. quantified the properties of asphalt mortars and asphalt mixtures containing municipal solid waste incineration (MSWI) fly ash as a filler, including the rheological properties, high-temperature properties, low-temperature properties, and water stability. The type of filler has little effect on the low-temperature properties of asphalt mortar. With an increased fly ash replacement rate, the high-temperature performance of the mix is enhanced. As the replacement rate of fly ash increases, the tensile strength and stiffness modulus of asphalt mixtures increase, and the low-temperature performance decreases. The addition of fly ash weakened the moisture stability of the asphalt mixture.

The uniformity of natural aggregate quality is difficult to control. The homogeneity of the material (e.g., density, hardness, etc.) and the shape characteristics of the aggregate (e.g., profile shape, angle, surface texture, etc.) control the homogeneity of the coarse aggregate quality. Li et al. [4] propose using 3D-printing technology to generate standard aggregate models, explore the feasibility of a direct preparation of standard aggregates using 3D-printing technology from the technical index and economic perspectives, and clarify the development direction of artificial aggregates. Through a series of indoor material tests, a cement-based material suitable for preparing high-strength artificial aggregates was developed, and a set of preparation processes was determined. Finally, the physical and mechanical properties of the natural aggregates were compared to verify the technical properties of the artificial aggregates and their engineering feasibility. The results demonstrated that photosensitive resin-based materials and ABS resin-based materials have excellent bending properties. However, they have weaker compressive strength and hardness compared to natural aggregates. Preparing resin-based artificial aggregates using 3D-printing technology is technically feasible, but its high cost severely limits the large-scale industrialization of on-site implementation. Cement-based materials are a low-cost and reliable alternative to the preparation of artificial aggregates using 3D printing technology. In this study, the optimal ratio of cementitious materials was proposed. The manufacturing procedures for cement-based artificial aggregates were improved and optimized. According to a comprehensive evaluation, the 3D shape of the prepared cementitious artificial aggregates has good agreement with the 3D morphology of natural aggregates.

Building demolition waste (BDW) is typically generated during the construction, maintenance, repair, rehabilitation, and demolition of buildings and other infrastructure. These wastes are heterogeneous and consist of different materials, such as concrete, brick, ceramics, wood, glass, plastics, soil, and gypsum-based construction materials, with bulky wastes (such as bricks and concrete) accounting for 70–80% of the total weight. Xiao et al. [5] investigated the feasibility and applicability of recycled BDW materials as unbonded granular fillers for pavement subgrade/subbase. Five different gradations were designed to represent the gradation changes caused by granular crushing, and through tests, data were obtained to propose an empirical mechanical prediction model for the modulus of elasticity by combining the gradation changes and stress states. This provided a technical reference and guidance to promote recycled BDW as an unbound granular material in pavement subgrade/subbase applications. In this paper, the authors investigated the effects of crushing-induced gradation changes on the shear strength and the resilient properties of BDW recycled aggregates through a series of monotonic triaxial compression tests and repeated load triaxial (RLT) tests. The authors propose a model for predicting the elastic modulus in combination with particle crushing. The model provides a useful guide for predicting the elastic properties of recycled BDW aggregates with significantly lower strength and friability. In addition, considering the high absorption ratio of recycled BDW aggregates, their water-sensitive properties are being further investigated to provide a more comprehensive understanding of the application of recycled BDW aggregates in pavement granular layers.

Constructing porous asphalt pavements is an effective way to promote sponge city infrastructure construction. The main asphalt binder used in porous asphalt is high-viscosity modified asphalt, which is overexposed to oxygen, temperature, water, and ultraviolet radiation, thus accelerating its aging damage, leading to the deterioration of asphalt pavement performance. In the study proposed by Zhang et al. [6], the aging characteristics of high-viscosity modified asphalt under multi-factor coupled environmental conditions were investigated based on infrared spectroscopy experiments. Finally, the aging kinetic equations of the high-viscosity modified asphalt based on a functional group index were established. The results demonstrated that high-viscosity asphalt is sensitive to complex aging factors through changes in chemical functional groups. 

The 3D concrete printing (3DCP) technique uses cementitious composite material deposited in layers of shapes with the help of computer design and large-scale 3D printers. Fly ash (FA), silica fume (SF), and ground granulated blast furnace slag (GGBS) are solid waste by-products from the energy and smelting industries, and cementitious composites using 3DCP technology use a combination of (FA), (SF), and (GGBS) to replace the cement binder. The study conducted by Wang et al. [7] aimed to investigate the effect of different steam-curing conditions on the mechanical properties of concrete using the 3DCP technique. Orthogonal experiments were performed to reduce the experimental workload and to obtain highly sensitive evaluation indices. In addition, coefficients were used to assess the anisotropy of the material quantitatively. Subsequently, the printed interlayer bonding properties were further analyzed. As a result, optimal steam-curing conditions for printed concrete were obtained for various mechanical properties and anisotropy. This study proposes that anisotropy coefficients and orthogonal tests can be used to evaluate the mechanical properties and anisotropy of concrete using the 3DCP technique when various steam-curing conditions are used for printed materials. Subsequently, optimal steam-curing conditions and interlayer bonding were demonstrated.

Wang et al. [8] investigated the mechanisms of water transformation and pore structure evolution of Cement-Stabilized Dredged Sediment (CDS) using the nuclear magnetic resonance (NMR) technique, and quantitative relationships between the macroscopic mechanical properties and the microstructure were established. A series of laboratory samples were prepared for different cement contents and maintenance periods. In addition, stress–strain curves were determined based on unconfined compression tests, and the influence of the water content parameters on strength development was quantitatively assessed from the perspective of stabilization mechanisms. Similarly, a mathematical model of the relationship between the hydraulic conductivity measured with a flexible wall penetrometer and the representative parameters of the pore size distribution was presented. Finally, the reliability of the NMR technique as a new method for studying the microscopic mechanisms of CDS strength and permeability evolution was further validated with scanning electron microscopy (SEM) and mercury-in-pressure porosity (MIP) tests.

Construction and demolition (C&D) waste can be utilized after incineration and activation. The properties of polypropylene fibers prevent cracking and inhibit brittle damage; moreover, polypropylene fibers mitigate sulfate attack. Zhang et al. [9] experimentally investigated the enhancement of cement-stabilized soil (CSS) properties with the introduction of treated C&D waste, polypropylene fibers, and sodium sulfate. For this, 10%, 20% and 30% of C&D waste were used as a substitute for cement. The dosages of polypropylene fiber and sodium sulfate were 1%, 2%, 4%, and 0.2%, and 0.4% and 0.8%, respectively. Uniaxial compressive strength (UCS) tests, flexural strength (FS) tests, and direct shear tests were conducted to examine the effect of coupling reinforcement on the mechanical properties of CSS. A backpropagation neural network (BPNN) and FR-tuned hyperparameters with the BAS algorithm were used to predict the UCS and FS properties of CSS based on 84 experimental results, which can be used as design guidelines for the future applications of such sustainable materials.

Zheng et al. [10] investigated the effect of carbonation on cementitious materials under waterlogged and saturated humidity environments by increasing CO_2_ concentration using an accelerated CO_2_ erosion tester that simulates the environment used in inflow tunnels. The results of the study can provide some theoretical and technical support for the design, construction, and safe operation of the diversion tunnel lining for the better sustainability of cementitious materials under carbonation environments.

Steel slag is a significant solid waste from steelmaking. The application of recycled steel slag in asphalt mixes is mainly hindered by the volume expansion after water absorption. Proper treatment methods must be proposed to solve the problem of water-induced volume expansion of steel slag and evaluate the submersion stability of the treated steel slag and its asphalt mixture. The mechanisms of oxalic acid and water erosion on the performance of steel slag and its asphalt mixture were analyzed by Huang et al. [11], and the suitability of steel slag and its mixture treated with oxalic acid was evaluated, providing engineering suggestions and theoretical guidance for the practical application of recycled steel slag in asphalt mixtures. In this study, steel slag was treated with a modified oxalic acid solution to inhibit the swelling and water erosion of steel slag and its asphalt mixture at different times before and after the treatment. Then, the basic properties of the steel slag and its asphalt mixture were tested by comparison. The mechanisms of oxalic acid and water erosion on the mixture properties of steel slag and its asphalt mixture were investigated. The potential of oxalic acid treatment of steel slag in asphalt mixes was examined.

Bio-oil can be recycled from plant and animal remains, restaurant waste oil, and discarded handicrafts. The use of biomass materials to modify asphalt is sustainable, and bio-oil can be added to asphalt/water emulsions as a penetrating agent, which is effective in increasing the penetration depth. Shi et al. [12] investigated the effect of emulsifier content on the stability of biomass asphalt/water emulsions based on molecular dynamics (MD) simulation techniques combined with macroscopic and microscopic experiments. The article investigated the effect of emulsifier dosage on the stability of biomass anionic bitumen/water emulsions from three perspectives, including interaction energy, interfacial layer thickness, and radial distribution function. The optimal emulsifier dosage was determined and verified by storage stability, particle size analysis and macroscopic and microscopic tests were conducted by infrared spectroscopy. Some waste shells have also been studied as biomass asphalt modifiers [13]. The road performance of modified asphalt with Trigonella mussel shell powder has been studied. The authors applied Trigonella mussel shell powder to modify asphalt in order to improve the road performance of asphalt pavement. The potential of shell powder as an asphalt modifier was found, and the high- and low-temperature properties and water stability of asphalt mixtures can be enhanced with the waste shell power modifier.

Recycling materials and most solid wastes can be treated and applied in infrastructure constructions for performance modification and enhancement. Natural resources and energy consumption can be significantly saved through these proposed green materials and innovative techniques, which would contribute to the development of infrastructure sustainability.

## References

[1] Xue X., Gao J., Wang J., Chen Y. (2021). Evaluation of High-Temperature and Low-Temperature Performances of Lignin–Waste Engine Oil Modified Asphalt Binder and Its Mixture. Materials.

[2] Xu F., Zhao Y., Li K. (2021). Using Waste Plastics as Asphalt Modifier: A Review. Materials.

[3] Zhao X., Ge D., Wang J., Wu D., Liu J. (2022). The Performance Evaluation of Asphalt Mortar and Asphalt Mixture Containing Municipal Solid Waste Incineration Fly Ash. Materials.

[4] Li W., Wang D., Chen B., Hua K., Huang Z., Xiong C., Yu H. (2022). Preparation of Artificial Pavement Coarse Aggregate Using 3D Printing Technology. Materials.

[5] Xiao Y., Kong K., Aminu U.F., Li Z., Li Q., Zhu H., Cai D. (2022). Characterizing and Predicting the Resilient Modulus of Recycled Aggregates from Building Demolition Waste with Breakage-Induced Gradation Variation. Materials.

[6] Zhang W., Li Q., Wang J., Meng Y., Zhou Z. (2022). Aging Behavior of High-Viscosity Modified Asphalt Binder Based on Infrared Spectrum Test. Materials.

[7] Wang B., Yao X., Yang M., Zhang R., Huang J., Wang X., Dong Z., Zhao H. (2022). Mechanical Performance of 3D Printed Concrete in Steam Curing Conditions. Materials.

[8] Wang S., He X., Cai G., Lang L., Ma H., Gong S., Niu Z. (2022). Investigation on Water Transformation and Pore Structure of Cement-Stabilized Dredged Sediment Based on NMR Technology. Materials.

[9] Zhang G., Ding Z., Wang Y., Fu G., Wang Y., Xie C., Zhang Y., Zhao X., Lu X., Wang X. (2022). Performance Prediction of Cement Stabilized Soil Incorporating Solid Waste and Propylene Fiber. Materials.

[10] Zheng J., Zeng G., Zhou H., Cai G. (2022). Experimental Study on Carbonation of Cement-Based Materials in Underground Engineering. Materials.

[11] Huang X., Yan F., Guo R., He H. (2022). Study on the Performance of Steel Slag and Its Asphalt Mixture with Oxalic Acid and Water Erosion. Materials.

[12] Shi S., Lin L., Hu Z., Gu L., Zhang Y. (2022). Study on the Stability of Bio-Oil Modified Prime Coat Oil Based on Molecular Dynamics. Materials.

[13] Fan G., Liu H., Liu C., Xue Y., Ju Z., Ding S., Zhang Y., Li Y. (2022). Analysis of the Influence of Waste Seashell as Modified Materials on Asphalt Pavement Performance. Materials.

